# Digital COVID Credentials: An Implementation Process

**DOI:** 10.3389/fdgth.2021.594124

**Published:** 2021-06-25

**Authors:** Mayssam Nehme, Laurent Kaiser, Philippe Gillet, Philippe Thevoz, Silvia Stringhini, Idris Guessous

**Affiliations:** ^1^Division of Primary Care Medicine, Geneva University Hospitals, Geneva, Switzerland; ^2^Faculty of Medicine, University of Geneva, Geneva, Switzerland; ^3^Division of Infectious Diseases, Geneva University Hospitals, Geneva, Switzerland; ^4^SICPA, Prilly, Switzerland; ^5^University Centre for General Medicine and Public Health, University of Lausanne, Lausanne, Switzerland

**Keywords:** digital, blockchain, COVID-19, decentralized governance, free movement, immunity, certificate, vaccination

Initial public health responses to the COVID-19 pandemic have focused on non-pharmaceutical interventions including stringent physical distancing measures, lockdowns, and restriction to free movement. This comes at significant costs however, both economically and socially (1, 2). As authorities begin to ease existing measures, governments are looking into specific alternatives to lockdown, such as phased mobilization of the economy (3), less stringent physical distancing measures, or immunity passports that would determine individual access or restrictions (4). Immunity passports vs. certificates differ in the rights related to their use and their issuing authority. Immunity passports have been cautioned against by the WHO and at international levels (5, 6) citing a lack of reliable interpretability of the presence or absence of COVID-19 antibodies, as well as ethical risks (7). With the advent of vaccines, these risks are potentially mitigated while other risks arise such as universal access to vaccination, and the debate around immunity passports is once again justifiably revived (8). COVID credentials could be an answer to facilitate some of the currently difficult scenarios in society and everyday life (travel, large gatherings, etc.). The need for a non-falsifiable solution is of utmost importance, especially with reports of fraud increasingly emerging (9).

Reflecting on the digital aspects of such a solution is important to ensure the implementation of adequate safeguards, display the right amount of information and use digital health systems to society's advantage. The European Union has recently published open source material detailing a potential trust framework and technical specificities that would be used in establishing a European Union Digital COVID Certificate that would be uniform and interoperable (10). COVID credentials taking into account vaccination, serology, PCR testing, and self-reported symptoms can employ algorithms to certify an individual's most recent COVID-related status. Certification would take into account results from pre-certified laboratories and pre-certified vaccination centers only, thus decreasing the prospect of false positive results and individuals inadvertently foregoing protective measures, putting themselves and others at risk (11). In addition, information could further assist individuals in making the right decisions and can also provide reminders to get tested or retested, vaccinated or re-vaccinated; which would also accommodate continually evolving aspects of the current COVID-19 pandemic and virus response. An example is setting reminders for individuals who received a vaccination to receive a booster shot, depending on the duration of the immune response (once defined), but also for individuals who received a specific vaccine to follow specific measures if a new variant turned out resistant to that vaccine. The presence of symptoms should also be part of the algorithm and could determine the need for fast-track testing or the implementation of isolation measures.

Here, we propose a very practical decentralized secured digital solution (Figure 1). The solution is securing the original data provided from a certified vaccination center, a certified laboratory or testing center. A digital security seal protects and guarantees the integrity of the data to be secured, through an unforgeable mathematical link between the hash of the data and the seal. To ensure the immutability, the digital security seal is timestamped on a blockchain. As the digital security seal contains only metadata, it guarantees privacy protection of the holder with personal and medical data only on the credential (QR code) itself. The blockchain is acting only as a secure “Trust Anchor,” in the form of an undisputable timestamp. Thus, no data are ever exposed or stored on the blockchain. Unlike the European Union Digital COVID Certificate, this solution does not need to handle the complex management of cryptographic keys, thus avoiding the risk of having some of these keys being compromised or stolen.

**Figure 1:**
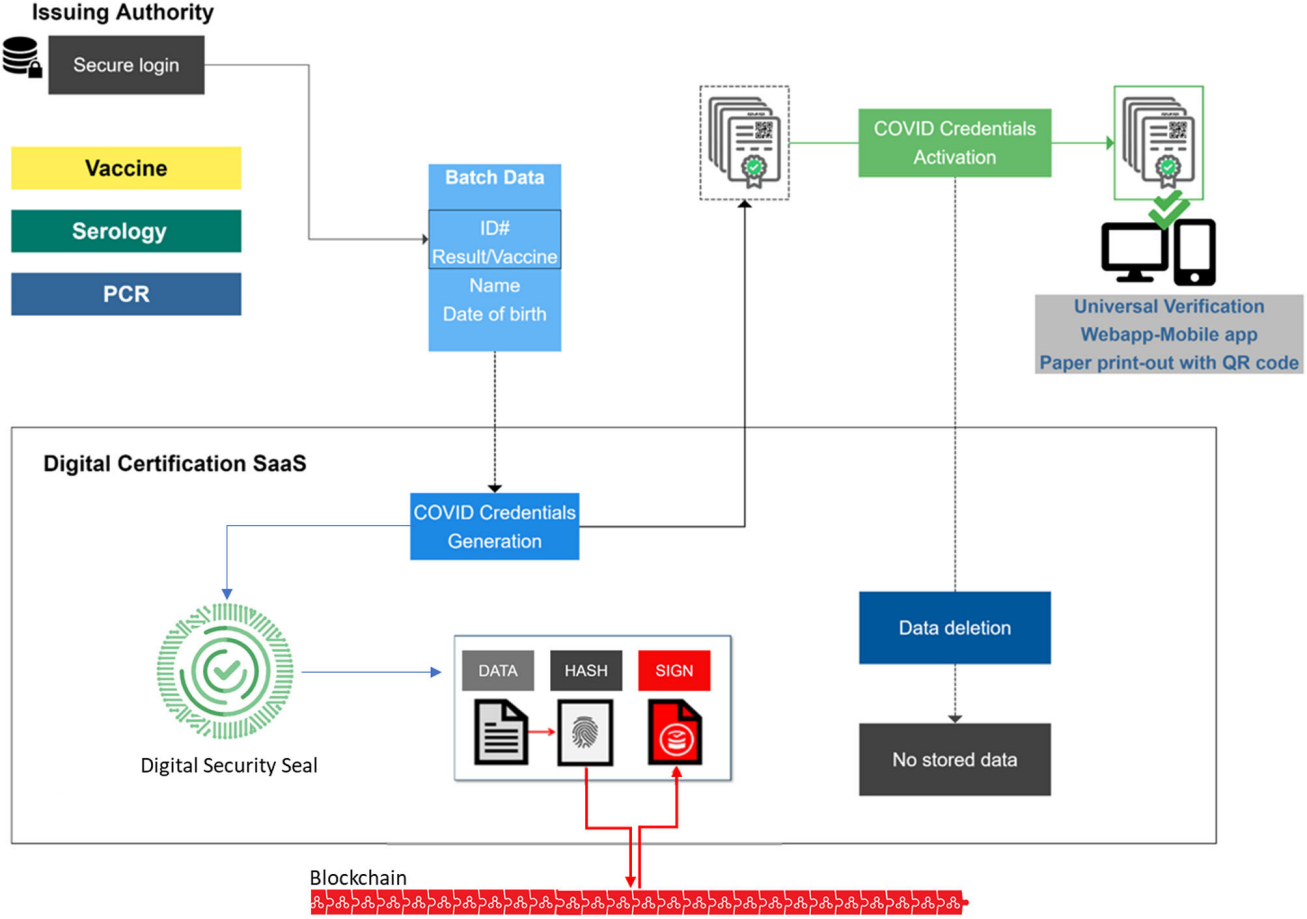
Digital process for COVID credentials.

The individual presents him or herself to the certified vaccination or testing center. His or her identity is verified (using an official ID) prior to testing, vaccination or determination of recovery. The information on vaccination status, or the test result or the recovery status is secured as COVID credentials. The COVID credentials consist of a certificate, secured by its QR code, containing the name of the person (previously verified), the medical information (vaccine, test result, recovery etc.) as well as the name and identification of the issuing authority. The COVID credentials are issued in batches (in the form of secured QR-codes) by the issuing authority (certified vaccination or testing center) using a Digital Certification SaaS. This Digital Certification SaaS is accessible online by the issuing authority only, with a secure login. Once the QR codes are generated, they are activated by the issuing authority and all information used to issue the credentials is deleted from the Digital Certification SaaS. This process reinforces the decentralized approach by removing the need for a central database that could be easily targeted, and safeguards are important to ensure only certified testing and vaccination centers are capable of issuing such credentials while respecting data protection and privacy regulations. The data remains in the issuing authority medical records (like any other laboratory or vaccination result and for a defined period of time if needed), enabling individuals to have their credentials re-issued when necessary (lost QR code for example). The secure QR code can be stored on an individual's phone or delivered as a print-out to reduce the digital divide. The secure QR code reduces the risk of forgery or tampering, and can be universally verifiable via a web-based portal or a mobile app, without the need to access a database containing personal or medical information. The individual has access to the web-based portal to verify his or her own credentials. The individual can choose to disclose information in specific contexts (airport control, access to a venue, nursing home, etc.) and interpretation of the result ensues, based on the context-related requirements (for example negative PCR within the last 72 h to enter a specific country vs. 24 h etc.). Individuals can selectively decide who to show this information to and how many identifying details to reveal depending on the context. Selective disclosure and decentralized information can further assist in preserving privacy and confidentiality. A digitally secured solution can also reduce the risk of loss, identity theft and forgery while ensuring accessibility, bidirectional information and the possibility to revoke the credentials or update the expiration information when needed. In order to ensure more universal access, a paper version of the digital certificate and QR code is also available. This paper version provides the same level of security as the digital one, as its content is certified via the QR code which can be universally verified with the same security as the digital credential. QR code verification acting as a digital unforgeable stamp remains a cornerstone of certification in order to avoid any fraud or falsification. The QR code verification can also be performed offline as the verification keys (digital security seals) can be periodically replicated locally on the verification device when connected.

## Conclusion

Immunity passports, certificates or COVID credentials will be increasingly at the forefront of medical and public policy discussions in the months and years to come. The adequate safeguards around a digital COVID credential should be discussed, and a non-falsifiable solution should be implemented especially if rights are linked to such credentials. The solution presented here provides a decentralized approach to databases as well as a secure certification process in line with the European Commission's recommendations (10). This solution also provides a secure approach, ensuring the integrity and validity of the information and respecting data protection regulations on privacy and confidentiality. The question of COVID credentials, now at the forefront, should be also be addressed at a policy level involving discussions between medical and public health actors, technology experts, ethicists and governing bodies. It is also of utmost importance to actively engage the public on the options and opinions connected with this issue in order to assess their trust and needs when proposing a digital health solution.

## Author Contributions

MN, PG, PT, LK, SS, and IG contributed to the writing of the manuscript. MN, PT, and IG contributed to the figures. All authors contributed to the article and approved the submitted version.

## Conflict of Interest

PG and PT has a patent WO2020011447 pending, and a patent WO2020030382 pending. SICPA has developed the CERTUS digital solution certificates with digital seal technology. The remaining authors declare that the research was conducted in the absence of any commercial or financial relationships that could be construed as a potential conflict of interest.
